# Motor directional tuning across brain areas: directional resonance and the role of inhibition for directional accuracy

**DOI:** 10.3389/fncir.2013.00092

**Published:** 2013-05-15

**Authors:** Margaret Y. Mahan, Apostolos P. Georgopoulos

**Affiliations:** ^1^Graduate Program in Biomedical Informatics and Computational Biology, University of MinnesotaMinneapolis, MN, USA; ^2^Department of Neuroscience, University of Minnesota Medical SchoolMinneapolis, MN, USA

**Keywords:** motor directional tuning, inhibitory mechanisms, movement direction, directional precision, motor resonance

## Abstract

Motor directional tuning (Georgopoulos et al., 1982) has been found in every brain area in which it has been sought for during the past 30-odd years. It is typically broad, with widely distributed preferred directions and a population signal that predicts accurately the direction of an upcoming reaching movement or isometric force pulse (Georgopoulos et al., 1992). What is the basis for such ubiquitous directional tuning? How does the tuning come about? What are the implications of directional tuning for understanding the brain mechanisms of movement in space? This review addresses these questions in the light of accumulated knowledge in various sub-fields of neuroscience and motor behavior. It is argued (a) that direction in space encompasses many aspects, from vision to muscles, (b) that there is a directional congruence among the central representations of these distributed “directions” arising from rough but orderly topographic connectivities among brain areas, (c) that broad directional tuning is the result of broad excitation limited by recurrent and non-recurrent (i.e., direct) inhibition within the preferred direction loci in brain areas, and (d) that the width of the directional tuning curve, modulated by local inhibitory mechanisms, is a parameter that determines the accuracy of the directional command.

## Introduction

Here, as in the thalamus and cortex, the conclusion is unavoidable: either there is an amazing degree of selectivity in the innervation of single neurons by afferent fibers or inhibitory and other synaptic mechanisms ensure that most inputs remain subthreshold

(Jones, 2007, p. 164]

In the quote above, Ted Jones referred to the high density of innervation of dorsal column nuclei by fibers traveling along the medial lemniscus, in what he called “enormous morphological convergence at all levels of the ascending somatosensory pathways” (Jones, 2007, p. 164), between periphery and cortex. The quote summarizes in a succinct way the puzzling fact that, despite all of this convergence, a remarkable specificity in receptive field size is present, which could be accounted for by “an amazing degree of selectivity … or inhibitory and other mechanisms” (Jones, 2007, p. 164). In 1984, Eb Fetz compared sensory and motor representations in the somatosensory and motor cortices, respectively, as follows: “… the receptive fields of sensory cortex cells represent the symmetric inverse of the muscle fields of CM (corticomotoneuronal) cells, insofar as cortical inputs are coded analogously to outputs. The symmetric representation of spatial fields of peripheral elements by cortical cells is clearly the result of convergent input connections to sensory cells and divergent output connections from precentral CM cells” (Fetz, 1984, p. 471). This comparison is misconstrued, for it does not make sense to equate *convergence* in the somatosensory cortex of peripheral inputs onto a single somatosensory cortical cell with *divergence* in the motor cortex from a single motor cortical CM cell to several motoneuronal pools. The comparison should refer to the same cortical feature, namely convergence or divergence for both cortices. In fact, the similarity between motor and somatosensory cortex holds at the same level of enquiry, namely the large convergence of thalamic and cortical inputs to both cortices (Darian Smith et al., 1990, 1993). The difference then lies in the content of information, which is manifested as a receptive field in the somatosensory cortex (Mountcastle et al., 1957) and as a directional tuning field in the motor cortex (Georgopoulos and Stefanis, 2007). In this sense, the somatosensory *tactile receptive field*, with (a) its central peak corresponding to that point in the skin where mechanical stimulation elicits the highest response, (b) the gradual reduction of activation as the stimulus is moved farther away from the “hot spot,” (c) the surrounding inhibition (Mountcastle and Powell, 1959; Mountcastle, 2005, p. 283), and (d) the gradual shift of the location of the receptive field in the skin, is qualitatively similar to the *directional tuning field* (Georgopoulos and Stefanis, 2007) with (a) its central peak corresponding to the preferred direction of movement, (b) the gradual reduction of activation with movements in directions farther away from the preferred direction, (c) the surrounding inhibition (Stefanis and Jasper, 1964a,b; Merchant et al., 2008; Georgopoulos and Stefanis, 2010), and (d) the gradual shift of the preferred direction in 3-D space (Naselaris et al., 2006b; Georgopoulos and Stefanis, 2007; Georgopoulos et al., 2007). As is the case with receptive fields being interconnected across sensory areas, an approximate topographic correspondence would interconnect directional tuning fields across various motor areas, which would account for the concurrent activation of these areas at the initiation and execution of a movement in a particular direction. In both sensory and motor systems, receptive field size and directional tuning width would be sharpened by recurrent and non-recurrent (i.e., direct) inhibitory mechanisms.

## Motor directional tuning

A basic finding in motor neurophysiology has been the discovery of directional tuning in space (Georgopoulos et al., 1982), namely the orderly variation of single cell activity with the direction of arm movement, such that activity is highest for a particular movement direction (the cell's “preferred direction”) and decreases progressively with movements farther and farther away from the preferred direction. Overall, the tuning is broad and is readily captured by a cosine tuning function. It is important that directional tuning exists for movements made in 2-D (Georgopoulos et al., 1982) as well as in 3-D space (Schwartz et al., 1988; Caminiti et al., 1990a). In fact, the 3-D tuning volume can be derived from a polar plot of a 2-D tuning curve by rotating the 2-D tuning curve around the axis of the preferred direction in 3-D space. In addition, directional tuning has been described for isometric force pulses (Georgopoulos et al., 1992; Taira et al., 1996) and for isometric ramp-and-hold forces (Sergio et al., 2005). In the latter study, the same motor cortical cells were studied under the isometric ramp-and-hold task and in a movement task: broad directional tuning was observed in both tasks but with varying degrees of congruence in the respective preferred directions.

The following also holds for directional tuning. (a) Given an ongoing tonic level of discharge in a given cell, directional tuning can occur due to graded increase in cell activity, combination of increase or decrease in activity, or graded decrease in activity. Although increase or decrease in cell activity cannot be assigned to changes in excitatory or inhibitory drive or a combination thereof, without intracellular recording, it is common to refer to the ongoing neural discharge as “net excitatory drive.” (b) Directional tuning is stable across different movement amplitudes wherever tested, including motor cortex (Fu et al., 1993), premotor cortex (Fu et al., 1993), supplementary motor area (Lee and Quessy, 2003), external and internal segments of the globus pallidus (Turner and Anderson, 1997), cerebellar cortex (Fortier et al., 1989), and deep cerebellar nuclei (Fortier et al., 1989). (c) The earliest changes in cell activity are also directionally tuned (Georgopoulos et al., 1982). And (d) the latency of onset of change in cell activity is also directionally tuned (Georgopoulos et al., 1982). These properties underscore the robustness of directional tuning which has now been described in various areas using fMRI (Fabbri et al., 2010).

The key parameter of the directional tuning curve is its preferred direction, for four main reasons. First, it is the only unique value in the curve; second, it encompasses the whole directional space, since it is distributed widely in 3-D space (Schwartz et al., 1988; Naselaris et al., 2006a); third, it is the basis for computing the neuronal population vector, a good predictor of movement direction (Georgopoulos et al., 1983, 1986, 1988; Schwartz, 1994), as the vectorial average of preferred directions weighted by the change in cell activity; and fourth, preferred directions are mapped in an orderly fashion in the motor cortex (Georgopoulos et al., 2007). Based on this mapping, we proposed (Georgopoulos and Stefanis, 2007) that the directional tuning curve comes about as the result of local, tangential interactions in the motor cortical circuit, with inhibition playing a crucial role (Georgopoulos and Stefanis, 2010; see also Merchant et al., 2012). Although these considerations capture the greater picture of directional tuning in the motor cortex, the details of motor cortical circuitry remains to be elucidated. A major advance in this field is the ability to record simultaneously from identified motor cortical cells *in vivo* (Sheets and Shepherd, 2011; Apicella et al., 2012) and thus explore relations based on cell type (e.g., inhibitory interneuron) and its projections (e.g., corticospinal or corticostriatal). This approach has already yielded novel insights into the local, orderly organization of motor cortical circuitry. An ultimate goal would be the combination of such an approach with behavior to elucidate the intricate relations between motor cortical circuitry and directional tuning.

## The ubiquitousness of motor directional tuning and its implications

Although first described in the motor cortex for arm movements in space (Georgopoulos et al., 1982), directional tuning has been found in practically all motor areas where it has been sought for, including premotor cortex (Caminiti et al., 1990b; Fu et al., 1993; Stevenson et al., 2012), human supplementary motor area (Tankus et al., 2009), parietal area 5 (Kalaska et al., 1983; Johnson et al., 1996), parietal area PEc (Battaglia-Mayer et al., 2001; Ferraina et al., 2001), area 7m of the medial wall (Ferraina et al., 1997), parieto-occipital area 6A (Battaglia-Mayer et al., 2000, 2001), external (GPe) and internal (GPi) segments of the globus pallidus (Turner and Anderson, 1997), motor thalamus (Inase et al., 1996), cerebellar cortex (Fortier et al., 1989), and deep cerebellar nuclei (Fortier et al., 1989). An important issue concerns how the directional tuning arises, i.e., what are the relevant synaptic interactions that underlie the shaping of single cell activity to a typically broad tuning function?

Remarkably, directional tuning curves are very similar in all areas and generally follow a cosine tuning function for 2-D space. In addition to the general shape of the tuning curve, all the other properties of directional tuning mentioned above are also observed in different areas. If we were to trace the sequence of events across brain areas following the onset of a visual stimulus instructing a movement in that direction, we would be impressed by the close temporal and directionally tuned engagement of the various areas, from the onset of the instructing stimulus to the onset of the movement. Although it is reasonable to assume a progression of directional information transmission from posterior (visual) to anterior (motor) areas, and, therefrom, bidirectionally to subcortical (thalamic, basal ganglia, and cerebellar) loops, it is remarkable that drastic changes in cell activity were observed, at the limit, as early as 40 ms following stimulus onset in a randomized movement direction task in the motor cortex (see, e.g., Figure 4 in Georgopoulos et al., 1982). These observations point to a strong directionally tuned co-activation among motor areas; we call this *directional motor resonance*. It is reasonable to suppose that this functional resonance emanates from the underlying pervasive anatomical, topographically organized, connectivity among the various motor areas and leads to the initiation of movement in the intended direction, an essential aspect of motor control (Hasan and Karst, 1989; Karst and Hasan, 1991a,b; Gottlieb et al., 1997). The orderly topographic connectivity constitutes one fundamental aspect of CNS motor control by which various brain areas become *directionally aligned*, so to speak. This seems to be matched by a ubiquitous local network mechanism that ensures spatial sharpening of directionality in each area, namely surround inhibition.

## Synaptic mechanisms underlying motor directional tuning

The locale of every area where directional tuning has been observed comprises both excitatory and inhibitory mechanisms. Although specifics differ widely (Table 1), there are certain common features that could account for the uniformity of directional tuning in various brain areas, as follows. First, excitatory and inhibitory mechanisms are both in play; second, both excitatory and inhibitory neurons receive inputs from local neurons as well as from external inputs; and third, the net effect of this interplay is transmitted further to other areas by outgoing projection cells. This net output is a directionally circumscribed motor signal that is transmitted to orderly, topographically connected recipient areas of a corresponding directional focus. The synaptic interactions within a specific area closely resemble those observed in the spinal cord when, e.g., a motor command arrives at a motoneuronal pool (Baldissera et al., 1981). The motor command typically impinges on (a) the targeted (anatomically) motoneurons, and, possibly, their agonists and synergists (depending on the context), (b) the Ia inhibitory interneurons to their antagonists, and (c) the Renshaw cells that provide recurrent inhibition (Renshaw, 1941; Baldissera et al., 1981; Windhorst, 1996). Now, this recurrent inhibition is distributed to (a) the alpha-motoneurons from which the Renshaw cells receive input, (b) the gamma-motoneurons of that pool, (c) the pools of the agonists and synergists, and (d) the Ia inhibitory interneurons to the antagonists (Baldissera et al., 1981). These actions have two major effects, namely (a) to limit the discharge of the motoneurons contacting the Renshaw cells, and (b) to sharpen the spatial extent of the excitatory motor command by exerting a flat inhibitory drive. In addition, Renshaw cells receive descending signals (Haase and van der Meulen, 1961; MacLean and Leffman, 1967; Fromm et al., 1977; Pierrot Deseilligny et al., 1977; Hultborn and Pierrot Deseilligny, 1979a,b; Hultborn et al., 1979a,b; Baldissera et al., 1981; Hultborn et al., 2004; Hultborn, 2006) that effectively can increase (if excitatory to Renshaw cells) or decrease (if inhibitory to Renshaw cells) the actions of the Renshaw cells on the various cell groups, as described above.

**Table 1:** **Summary of synaptic inputs to areas with known motor directional tuning**.

	**Directional tuning**	**Local inhibition**	**Excitatory external inputs**	**Inhibitory external inputs**
Cerebral cortex	Yes	Yes	Yes (from thalamus and cortex)	No
Motor thalamus	Yes	Yes	Yes (from cortex and deep cerebellar nuclei)	Yes (from globus pallidus)
Globus pallidus	Yes	No	Yes (from subthalamic nucleus and cortex)	Yes (from putamen)
Cerebellar cortex	Yes	Yes	Yes (from mossy and climbing fibers)	No
Deep cerebellar nuclei	Yes	Yes	Yes (from mossy and climbing fibers)	Yes (from cerebellar cortex)

Interestingly, qualitatively similar arrangements are found in all motor areas of the central nervous system, including the cerebral cortex, thalamus, cerebellar cortex, deep cerebellar nuclei, and basal ganglia, in the sense that inhibitory mechanisms play a major role in shaping the local activation landscape. However, there are differences in the origin and distribution of the inhibitory drive, as follows. In the cerebral and cerebellar cortex, all inhibitory mechanisms are local; there is no direct inhibitory input from the outside. By contrast, in the globus pallidus, all inhibitory input is external, arriving from the striatum. And in the thalamus and DCN, the situation is mixed, in that there are both local inhibitory neurons but also external inhibitory inputs arriving from the globus pallidus and cerebellar cortex, respectively. Excitatory inputs arrive from several sources to all areas above, namely from (a) all external inputs to the cerebral cortex (thalamocortical, corticocortical), (b) mainly the subthalamic nucleus to the globus pallidus, (c) corticothalamic and deep cerebellar nuclei inputs to the thalamus, (d) mossy and climbing fibers to the cerebellar cortex, and similarly to the (e) deep cerebellar nuclei (Uusisaari and De Schutter, 2011). In spite of this diversity of excitatory-inhibitory mechanisms, cells in all areas above show broad directional tuning (see above). *Thus, a remarkable functional relation to movement direction is preserved across areas*. The most likely explanation lies in the pervasive, albeit rough, topographical correspondence in anatomical connectivity and in the parallel presence of seemingly non-specific (Fino and Yuste, 2011; Fino et al., 2012) inhibitory mechanisms. This arrangement preserves (a) a correspondence of concurrent activation of neurons with similar preferred directions across various brain areas, and (b) limits the spatial extent of activation, resulting in the directional tuning.

At this point, a consideration of the diverse types of inhibitory neurons and their possible functional impact is in order. This diversity has been stressed as implying a correspondingly diverse specificity in inhibitory action (Burkhalter, 2008); in contrast, a different view has been advanced (Battaglia et al., 2013), namely that such diversity does not have to translate necessarily to functional specificity and that, instead, a *functional degeneracy* is in play, namely that different neuronal populations can contribute and/or cooperate to the same function, as is the case, for example, for neurovascular coupling which can be mediated by multiple vasoactive messengers produced by different cell types (see Battaglia et al., 2013 for a more detailed discussion). An appreciable diversity in biochemical and histochemical cell properties exists for the Renshaw cells and other inhibitory interneurons in the ventral horn of the spinal cord (Fyffe, 1991; Alvarez et al., 1997; Hellstrom et al., 1999; Carr et al., 2000, 2001; Geiman et al., 2002; Hughes et al., 2013), and, yet, the overall function of inhibition exerted by Renshaw cells or Ia interneurons is relatively well-understood. Therefore, we agree with Battaglia et al. (2013) in their summary that the cell type diversity most probably reflects functional degeneracy (Edelman, 1987), “defined as the ability of heterogeneous elements to perform the same function (in this case, inhibition). Beyond *redundancy*, occurring when a given function is achieved by replicating homogeneous elements, *degeneracy* confers higher robustness through adaptability. Indeed, heterogeneous elements can react differently in different contexts providing a considerable margin of safety over a wide spectrum of conditions” (Battaglia et al., 2013, p. 19). In summary, there could very well be a meaningful function of cell type diversity within a neural circuit but this may not be necessary reflected at the “bird's eye view” level of assessing excitatory-inhibitory interactions within the circuit. For example, the view of a forest in the middle of a valley, and their borders, are clear-cut from an overflying helicopter but this does not preclude the fact that the forest may be composed of a variety of trees and the valley of a variety of grass and bushes. The internal composition of the forest and the valley perhaps may well-serve specific purposes but, for other purposes, the forest-valley distinction is clear-cut and does not depend on the respective internal compositions.

Now, there are additional aspects of directional motor control, e.g., strength and accuracy of a movement. It is worth exploring how the considerations above would be brought to play on these issues. For that purpose, it is instructive to examine the case of Renshaw cells in more detail. As mentioned above, descending inputs impinge on Renshaw cells to excite or inhibit them (see above): in the former case, net excitation is stronger and more widespread at the motoneuronal level, whereas in the latter case it is weaker and more spatially limited. Now, it has been shown in humans that such effects are meaningfully in operation. For example, weak tonic muscle contractions were associated with increased recurrent inhibition, whereas strong contractions were associated with decreased inhibition (Hultborn and Pierrot Deseilligny, 1979a). Thus, central control of Renshaw cell activity has been linked to a role of recurrent inhibition in spatial sharpening of the motor command to the spinal cord and in adjusting the magnitude of its effect, depending on the task.

It should be noted that the task-related modulation of Renshaw cell activity should be exerted independently of the motor command itself (Koehler et al., 1978). A separate control of motoneuronal and Renshaw cell excitability is almost necessary to achieve, for example, the strong and extensive excitation of the motoneuronal pool and its agonists: if the same, strong motor command was also applied indiscriminately to the Renshaw cells, its strength on, and extent among, motoneuronal pools would be limited by the increased recurrent inhibition. And the same considerations apply for the opposite case, namely the development of weak motoneuronal activations coupled with high spatial sharpening. Indeed, an independent control of Renshaw cell activity by descending inputs has been well-documented (Koehler et al., 1978).

## The role of inhibition in motor directional tuning

It is tempting, then, to speculate on a possible correspondence between Renshaw inhibition in the spinal cord and inhibitory mechanisms in the cerebral cortex with respect to shaping the motor command. In the Renshaw cell case, the shaping of the motor command refers to the distribution and strength of activation of various motoneuronal pools involved, whereas in the case of cortical inhibition the shaping of the motor command refers to the directional tuning, in preparation of the upcoming movement. Of course, the two commands are intimately interrelated (see next section). (See also Georgopoulos and Grillner, 1989, for a general discussion of similarities between motor cortical and spinal mechanisms in motor control.) Now, there are two features that are similar in both inhibitory mechanisms: first, the distribution of inhibition is basically non-specific (Baldissera et al., 1981; Fino and Yuste, 2011; Fino et al., 2012), and second, inhibitory cells are subject to control independently of other neighboring cells, e.g., motoneurons in the spinal cord or pyramidal cells in the cortex. As mentioned above, the role of Renshaw cell inhibition in weaning out weak excitation and spatially delimiting activation, is well-established (Baldissera et al., 1981). Similarly, the central role of inhibition in directional tuning in the cortex is widely accepted (see Merchant et al., 2012 for a review). However, a task-related control of the inhibitory drive has not been brought up in discussions of cortical inhibition, although it has been established with respect to Renshaw inhibition (Hultborn and Pierrot Deseilligny, 1979a). We would like to propose the existence of such a task-related function for the cortical inhibitory drive, namely *to change the width of the directional tuning curve, dependent on the directional accuracy of movement required*. Specifically, we hypothesize that the tuning width will be adjusted to implement the initiation of directionally accurate movements: *the more directional accuracy is required, the more narrow the directional tuning curve will be*. We examined the relation between directional accuracy and directional tuning width in a simulation, as follows.

**Figure 1:**
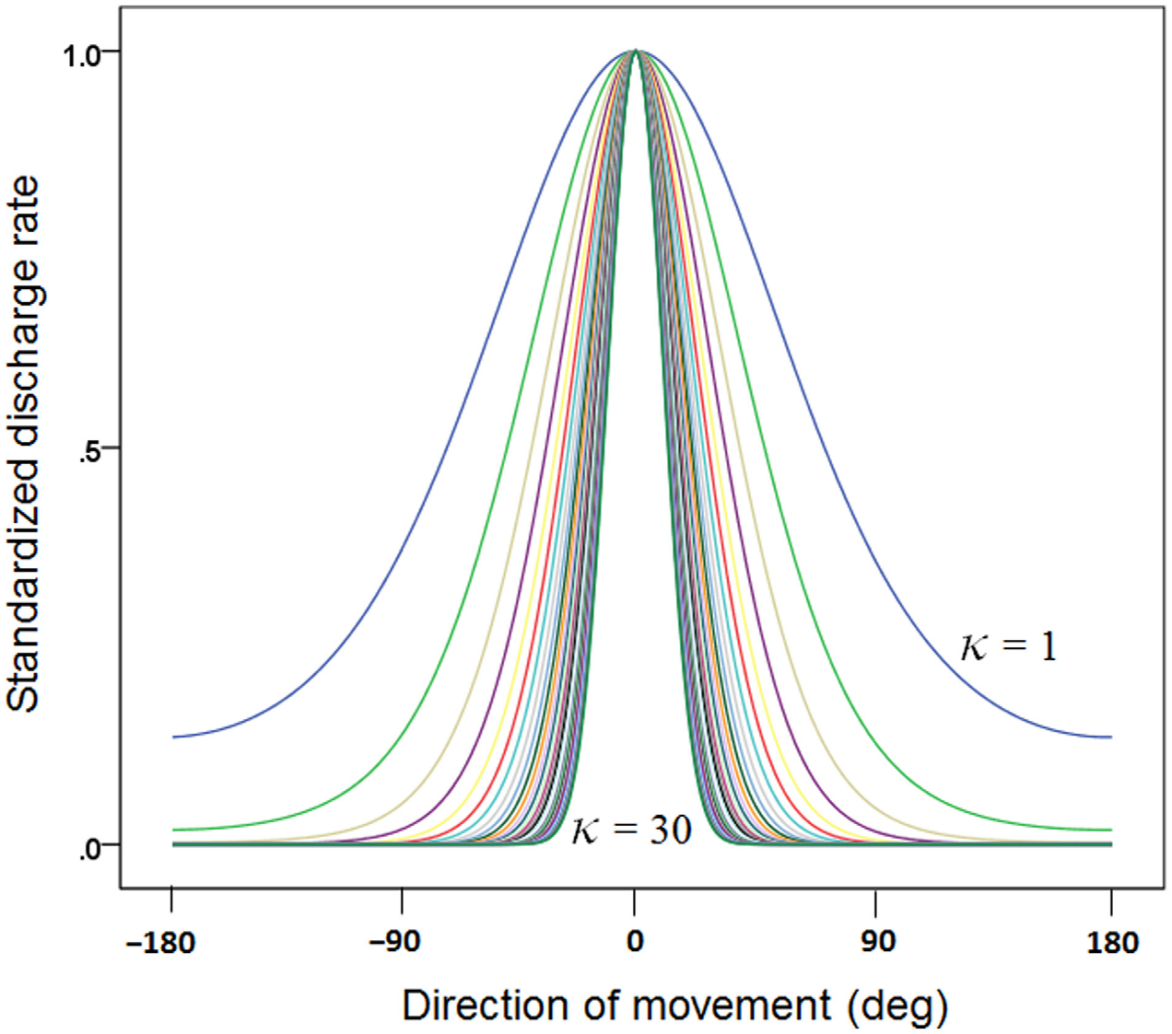
**Plot of 30 directional tuning curves generated using Equation 1 (see text), one for each one of 30 κ values {1, 2, 3, …, 28, 29, 30}.** Each curve is standardized to its maximum.

**Figure 2:**
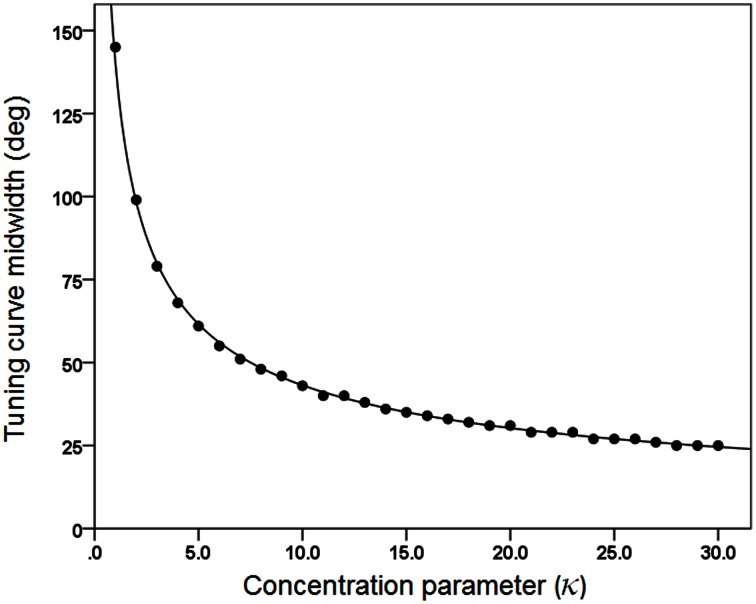
**The midwidth of the tuning curve is plotted against κ.** The fitted line is a power function fit (*R*^2^ = 0.999). *N* = 30.

- We generated 2-D directional tuning curves using the “circular normal distribution” (Amirikian and Georgopoulos, 2000):
  (1) $$d=e^{\kappa \cos(\theta -\mu )}$$
  where *d* is the discharge rate of a hypothetical cell for a movement in direction μ, θ is the cell's preferred direction, and κ is the concentration parameter (similar to the inverse of the variance in the normal distribution).
- The value of *d* was standardized to the peak of the curve:
  (2) $$d^{\prime }=\frac{d}{\max(d)}$$
- We generated 30 basic tuning curves, one for each κ taking systematically the values {1, 2, 3,…, 28, 29, 30}. These curves are shown in Figure 1. We also calculated the tuning curve midwidth, which varied from 145 (κ = 1) to 25° (κ = 30). The midwidth was a power function of κ (Figure 2).
- Next, we generated 10,000 tuning curves for each κ value, with θ chosen at random from a uniform distribution in the range of −180 to +180°, and with 1° resolution. (e) We fixed μ = 0 and calculated 1000 population vectors using *d*' as a weight. (f) For each population vector, we calculated the angle ω between its direction and the direction of movement (at 0) and the resultant *R* which corresponds to the length of the population vector:
  (3) $$R={\left(\sin{\left(\omega \right)}^{2}+\cos{\left(\omega \right)}^{2}\right)}^{1/2}$$
  The angle ω is signed (i.e., clockwise or counterclockwise). The average ω′ of the absolute value of the angle (over 1000 population vectors) is an estimate of the overall directional variability of the population vector (“variable error”):
  (4) $$\omega^{\prime }=\frac{1}{1000}\sum \left|\omega \right|$$
  whereas the average ϕ of the signed angle is a measure of directional bias of the population vector (“constant or signed error”):
  (5) S=∑sin(ω)
  (6) C=∑cos(ω)
  (7) $$\varphi =\text{ atan }\left(\frac{S}{C}\right)$$

**Figure 3:**
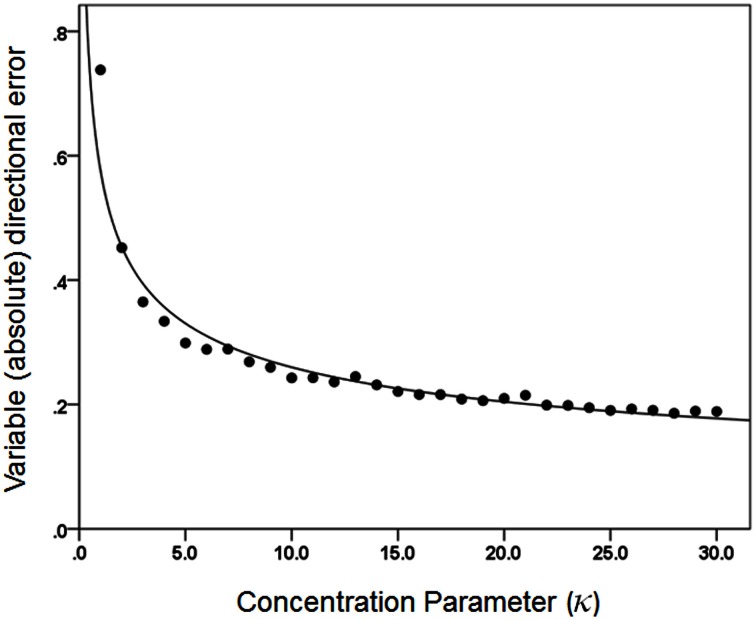
**The average directional variable error of the 1000 population vectors is plotted against κ.** The fitted line is a power function fit (*R*^2^ = 0.957). *N* = 30.

**Figure 4:**
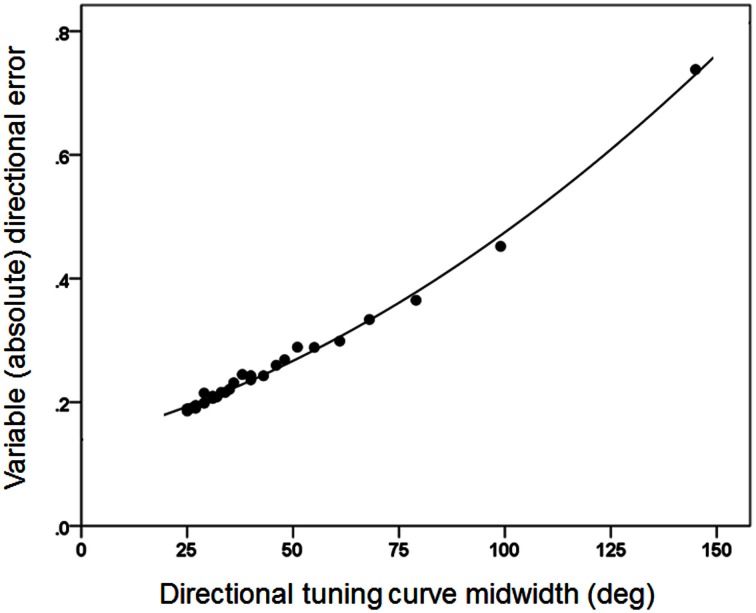
**The same variable error as in Figure 3 is plotted against the directional tuning curve midwidth.** The fitted line is a quadratic fit (*R*^2^ = 0.995).

Figures 3, 4 plot the variable error ω′ of the population vector against the concentration parameter κ and midwidth of the tuning curve, respectively. It can be seen that the variable error of the population vector increases as a power function with decreasing κ (Figure 3) and as a quadratic function of tuning curve midwidth (Figure 4). As expected, the constant, signed directional error of the population vector did not vary significantly from 0 for any κ (data not shown). These results demonstrate that, first of all, the width of the directional tuning curve is an important variable that can affect significantly and systematically the variation in direction of the population vector, a good predictor of the upcoming movement (Georgopoulos et al., 1983, 1984, 1986, 1988). Assuming that recurrent and non-recurrent (i.e., direct) inhibitory mechanisms are basic means by which the directional tuning width is manipulated, their control, in turn, provides a fundamental mechanism for initiating and controlling movement in space to desired specifications, according to a particular task. That way, the role, contribution and control of central inhibitory mechanisms is at last aligned with those known since long ago for the Renshaw inhibition in the spinal cord, since a fundamental aspect of both is their task- or context-dependent modulation.

Finally, the results above have additional implications for theoretical considerations and some findings in the literature. For example, it has been tacitly taken for granted that directional tuning is associated with a cosine function, since this function has fitted experimental data well. In the light of the results above, it is possible to attribute the wide presence of cosine tuning (i.e., a tuning midwidth of 90°) to the use of 8 movement directions (every 45°) used in those experiments (Georgopoulos et al., 1982, and all studies in other brain areas). Interestingly, when 20 directions were used (every 18°), the tuning midwidth was found to be ~50° (Amirikian and Georgopoulos, 2000). This issue awaits further, systematic investigation.

Finally, our simulations above revealed another correspondence to the Renshaw cell regulation and to the motor field in general, namely the mechanisms underlying the speed-accuracy tradeoff. With respect to the Renshaw cell regulation, it was mentioned above that an increased supraspinal excitatory drive on the Renshaw cells results in two, combined effects, namely (a) spatially limiting the excitatory drive on the agonist motoneurons, and, therefore, (b) reducing the overall motor output, at the same time. This can be considered as the spinal neurophysiological basis for the well-known speed-accuracy tradeoff (Fitts, 1954) which states that movement time is a log function of movement amplitude and target width, as follows:

(8) $$MT=a+b\log_{2}\left(\frac{2A}{W}\right)$$

where *MT* is the movement time, *A* is the amplitude of the movement, and *W* is the width of the target, and *a* and *b* are regression coefficients. MacKenzie (1992) derived a more accurate equation:

(9) $$MT=a+b\log_{2}\left(1+\frac{A}{W}\right)$$

For a movement of unit amplitude, Equation 9 becomes:

(10) $$MT=a+b\log_{2}\left(1+\frac{1}{W}\right)$$

Now, for a fixed movement of unit amplitude, the length of the population vector *R* (Equation 3) can be considered a velocity signal, such that:

(11) $$MT^{\prime }=\left(\frac{1}{R}\right)$$

Also, the variable directional error ω of the population vector can be considered as the target width. Substituting in Equation 10, we get:

(12) $$MT^{\prime }=\left(\frac{1}{R}\right)=a+b\log_{2}\left(1+\frac{1}{\omega }\right)$$

We evaluated this model in the sample of 1000 population vectors generated by varying the width of the directional curves, as described above. There was a good fit (*r* = 0.317). In contrast, the fit for a linear model (i.e., without the log transformation in Equation 12) was poor (*r* = 0.178). These results demonstrate that the speed-accuracy tradeoff (Fitts' law) holds at the level of the population vector, which brings it into alignment with the Renshaw cell—motoneuronal output interactions discussed above. Specifically, in both cases, an increase in inhibitory drive will result in a more spatially (directionally) accurate but weaker (slower) motor output.

**Figure 5:**
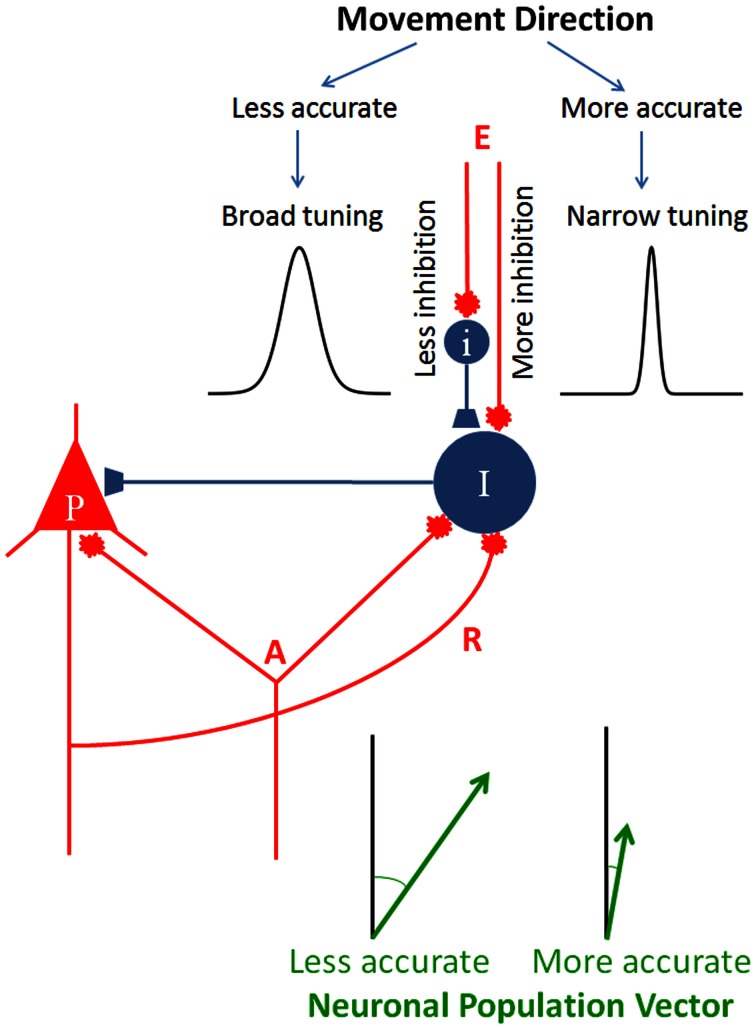
**Schematic diagram to illustrate the hypothesis of directional accuracy via a variably tuned circuit.** Red and blue terminals indicate excitatory and inhibitory synapses, respectively. P, pyramidal cell; I and i, inhibitory interneurons; A, afferents impinging on both the pyramidal cell and inhibitory interneuron; R, recurrent collateral. The two tuning curves were chosen from those shown in Figure 1 to illustrate two different tuning widths. The lengths of the two population vectors differ to illustrate the speed-accuracy tradeoff (see text for details).

This idea of specifying the directional accuracy of the movement by modulating the inhibitory drive to the circuit is illustrated in Figure 5. An elementary cortical circuit consists of a pyramidal cell (P) and two inhibitory interneurons (*I*). There are three kinds of inputs to those cells: (a) afferents (*A*) which impinge on both pyramidal and inhibitory cells, (b) recurrent collateral (*R*) from the pyramidal cell on to the inhibitory interneuron, and (c) external inputs (*E*), private to inhibitory interneurons. Inputs *A* and *R* are standard in cortical microcircuitry literature (DeFelipe and Jones, 2010; Douglas and Martin, 2010; Georgopoulos and Stefanis, 2010; Markram, 2010) but input E is not. This kind of input is key to our hypothesis which posits a private modulation of the inhibitory drive for the control of tuning width, exemplified by the two tuning curves shown in Figure 5. To our knowledge, such an input has not been looked for. In fact, there has not been a systematic study of the inputs to inhibitory interneurons, beyond recurrent collaterals and common afferents. As Douglas and Martin (2009) succinctly pointed out, “While past research has focused almost exclusively on the output of the inhibitory neurons, one crucial aspect of future circuit analysis is to determine the source of the inputs to the inhibitory neurons and to then combine this with knowledge of the dynamics of spiking patterns and synaptic plasticity” (Douglas and Martin, 2009, p. R402). Figure 5 illustrates two separate mechanisms controlling inhibitory drive of interneuron *I*, one purely excitatory and another inhibitory, exerted through interneuron *I*, although a continuous modulation of the excitatory input alone would be sufficient as well.

All of the discussion above was centered on directional control. The next question, therefore, is: why is movement direction such a pervasive motor parameter? We discuss this question next.

## The pervasiveness of motor direction

The commonest use of the arm is to move the hand from one point to another to acquire objects of interest. The initial and final positions of the hand in extrapersonal space define a “visuomotor” vector. Motor directional tuning means that neural activity varies in an orderly fashion with the direction of this vector, relative to its origin. A neural population signal (neuronal population vector) calculated based on changes in cell activity from a set level predicts accurately the direction of the visuomotor vector above (Georgopoulos et al., 1983, 1984, 1986, 1988) in any area it has been calculated (Georgopoulos et al., 1988; Fortier et al., 1989; Kalaska et al., 1989; Caminiti et al., 1990a,b, 1991; Truccolo et al., 2008). In both cases, regarding either single cell activity or the population vector, the direction of movement is the central driving motor variable. The directional tuning was discovered at about the same time that emphasis was given to the movement trajectory (kinematics) as a key aspect of planning multijoint movements in space (Georgopoulos et al., 1981; Soechting and Lacquaniti, 1981; Abend et al., 1982; Viviani and Terzuolo, 1982), an idea that was further developed and amplified in several studies during the 1980's. Later on, the importance of the direction of movement within the muscles-and-torques domains became apparent. In a series of papers (Hasan and Karst, 1989; Karst and Hasan, 1991a,b). Zia Hasan and collaborators drew attention to the importance of direction for the initiation and control of multijoint arm movements by analyzing patterns of changes in electromyographic (EMG) activity in muscles engaged in such movements. Those studies identified the movement direction from the initial to the final position, relative to the forearm, as the key variable to which EMG sign, intensity, and muscle selection relates to. Two additional studies also identified movement direction as a key variable, from two very different perspectives. In one study (Gottlieb et al., 1997), subjects made reaching movements in the sagittal plane in different directions, from various starting positions and of various amplitudes. It was found that, for movements in almost every direction, the dynamic components of the muscle torques at both the elbow and shoulder were related almost linearly to each other, and that the relative scaling of the two joint torques varied continuously and regularly with movement direction, in spite of the complex non-linear dynamics of those multijoint movements (see also Shemmell et al., 2007). These findings underscore the importance of movement direction in multijoint torque coordination. The other study (Worringham and Beringer, 1989) discovered a fundamental relation between direction defined in visual field coordinates and direction of arm movement relative to the forearm. By testing subjects in postures that altered the relative positions of the head, trunk, and arm, it was found that subjects planned and executed movements much more rapidly when the direction of movement of the visual target was used to instruct the direction of arm movement relative to the forearm, rather than relative to the trunk or relative to the surroundings. The blending of visual, hand- and eye-related motor signals has been amply documented in neurophysiological studies; in addition, there is a congruence in directional tuning between the two main effectors in eye-hand coordination, namely hand and eye movements (see Battaglia-Mayer and Caminiti, 2002 and Caminiti et al., 2010 for reviews). Remarkably, the interplay of these factors with respect to the direction of visually instructed eye or hand movements has been well-documented and delineated in a series of pioneer studies by Roberto Caminiti and collaborators, spanning an amazing range of cortical areas, from area 7 m to the premotor cortex (see Battaglia-Mayer and Caminiti, 2002). In fact, these investigators have proposed that degradation of the congruence in the directional tuning of neural activity to hand and eye movements (“global detuning”) might be the neural mechanism underlying the clinical syndrome of optic ataxia, essentially a movement direction apraxia.

All of the studies above point to the direction of movement as a fundamental variable in arm motor control (Georgopoulos, 1996), both intrinsically and in a visuomotor setting. In fact, the fundamental role of motor direction extends beyond movement itself to isometric force control. In a series of experiments using a strictly isometric manipulandum (Massey et al., 1988), we found that visually instructed isometric force trajectories made by human subjects possessed all the known properties of multijoint movement trajectories, namely obeying the 2/3 power law and being piecewise planar (Massey et al., 1991a,b, 1992; Pellizzer et al., 1992). These findings point to the spatial aspects of the motor trajectory being at the heart of the matter, and not the coordination of moving limbs. This idea gained further support by the results of a neurophysiological experiment in which we recorded single cell activity in the motor cortex of monkeys while they produced force pulses in visually specified directions in the presence of static loads applied in various (steady) directions (Georgopoulos et al., 1992). We found that single cell activity was directionally tuned to the net force of the isometric pulse produced, and the neuronal population vector pointed in the direction of that net force. Steve Wise lucidly and succinctly discussed the implications of this finding for neural motor control (Wise, 1993). Finally, a broad directional tuning to isometric ramp-and-hold forces has also been described (Sergio et al., 2005). These investigators also recorded the activity of the same motor cortical cells in a movement task. A broad directional tuning was observed in that task as well but the preferred directions differed frequently, especially when calculated along different time bins. These results underscore the richness of the time-varying relations between motor cortical activity and motor parameters, depending on the task and its context.

In the context of this review, there are two points of interest. The first point emphasizes the importance of the spatial aspects of motor direction, in the absence of limb motion, as discussed above. The second point is more subtle but as important, namely that, during force pulse production, motor cortical activity related to the *change* in force, which was in the visually instructed direction. This result is relevant with respect to the findings above by Hasan and collaborators on the importance of the direction of arm movement *relative to the forearm* for specifying the pattern of muscle activation. Qualitatively, the static load against which the monkey was holding before the initiation of the force pulse could be compared to the maintenance of a fixed forearm posture before the initiation of movement. If so, motor cortical activity would indeed relate to the direction *relative to the static load*. In fact, the results of crucial neurophysiological experiments have addressed satisfactorily this issue. Specifically, when the neuronal population vector was calculated as a time-varying measure using as weights on single cell contributions the *difference* between their ongoing activity and the steady-state activity they had during the preceding control period (steady holding), the population vector direction was an unbiased estimate of the instantaneous direction of the movement trajectory (Georgopoulos et al., 1984, 1988; see also Georgopoulos, 1995). Even more interestingly, when the population vector was calculated under conditions of different initial arm positions, it was again an unbiased estimate of the direction of movement, even though the preferred direction of individual cells had shifted (Caminiti et al., 1990a,b, 1991).

## Concluding remarks

In summary, motor directional tuning is a fundamental aspect of brain control of movement. Beyond the scientific value of this finding, directional tuning is at the base of current neuroprosthetic applications (Velliste et al., 2008; Collinger et al., 2013; Hochberg et al., 2012). There are many different reasons why “direction” in space is so influential in driving cell activity in so many and diverse brain areas. There are three important points: first, various “directions” are quite congruous with each other, e.g., visually specified direction, direction of a saccadic eye movement, direction of reaching, direction of isometric force pulse; second, “direction” is fundamentally a spatial attribute, hence it cuts across specific instantiations above; and third, it so happens that the joint torques and EMG activity vary in an orderly fashion with movement direction. The net result of all of this is that “everywhere you look, there is direction,” which means that cell activity in many different areas will vary with direction, but *not all of them for the same reason*. What saves the day is the pervasive *congruence* among the various “directions” and the covariation of multijoint limb kinetics and muscular activity with movement direction. This state of affairs enables a distributed *directional resonance* across diverse brain areas, each one of which is concerned with its own “direction” or directional blend(s) thereof. Of course, the directional congruence (and resulting resonance) comes from the rough anatomical/topographic correspondence in connectivity, which makes all of the areas involved to become concurrently active.

Another major question is why directional tuning is broad and so similar across diverse brain areas. We propose that this reflects two main factors, namely (a) the presence of recurrent and non-recurrent (i.e., direct) inhibition present in all these areas under different disguises, and (b) the accuracy demands of the specific task. Both of these factors have to do with the inhibitory drive which limits the spatial extent of activation. In addition, an independent excitatory or inhibitory control of local inhibitory mechanisms can modulate the width of directional tuning which, in turn, determines the variability of the direction of the motor command, as illustrated in Figure 5. Thus, movements of desired directional accuracy can be planned by varying the degree of activation of the local inhibitory interneurons, a mechanism analogous to the known descending modulation of Renshaw cell inhibition in task-related contexts. The findings of our simulation studies presented above document and quantify this relation.

### Conflict of interest statement

The authors declare that the research was conducted in the absence of any commercial or financial relationships that could be construed as a potential conflict of interest.
